# Enhanced Computed Tomography and ^18^F-fluorodeoxyglucose Positron Emission Tomography/Computed Tomography in the Uncommon Histiocytic Sarcoma of Small Intestine Arising after Gastric Large B-Cell Lymphoma

**DOI:** 10.3390/diagnostics13203189

**Published:** 2023-10-12

**Authors:** Miju Cheon, Jang Yoo, Hae Su Kim, Miji Lee

**Affiliations:** 1 Department of Nuclear Medicine, Veterans Health Service Medical Center, Seoul 05368, Republic of Korea; 2 Division of Hematology-Oncology, Department of Internal Medicine, Veterans Health Service Medical Center, Seoul 05368, Republic of Korea; 3 Department of Pathology, Veterans Health Service Medical Center, Seoul 05368, Republic of Korea

**Keywords:** histiocytic sarcoma, FDG, PET/CT, hematologic neoplasm

## Abstract

Histiocytic sarcoma (HS) is an extremely rare and aggressive malignant neoplasm of hematopoietic origin that shows morphologic and immunophenotypic evidence of histiocytic differentiation. In approximately 25% of the cases, presumed transdifferentiation of a preexisting hematolymphoid disorder can be demonstrated. Various extranodal sites, particularly the gastrointestinal tract, soft tissue, skin, and spleen, can be involved. Enhanced CT and FDG PET/CT findings of extranodal histiocytic sarcoma have been barely reported. We present a case with extranodal HS originating in the small intestine after gastric large B-cell lymphoma, mistaken for prostate cancer metastasis in a 76-year-old man.

A 76-year-old man complaining of a left inguinal mass was referred to our hospital. He had a history of gastric lymphoma treated with systemic chemotherapy 17 years ago. A gastric ulcer was discovered in a gastroscopy that started with symptoms of stomach bloating, and diffused large B-cell lymphoma was proven in a biopsy through an endoscope. Afterward, the patient underwent six cycles of chemotherapy with R-CHOP, and the disease was remitted entirely, with no signs of recurrence during follow-up examinations for several years. Also, he had prostate cancer treated with radical prostatectomy two years ago. The lowest PSA level after surgery was 0.061 ng/mL, which was maintained between 0.061 ng/mL and 0.230 ng/mL until the last three months. Contrast-enhanced abdominal computed tomography (CT) for evaluating the left inguinal mass revealed not only a left inguinal hernia but also unexpectedly enhanced infiltrative soft tissue mass and nodules in the right retroperitoneal fascia, right psoas muscle, and terminal ileum (Figure 1A–D). In this context, metastatic lesions from prostate cancer were suspected because the serum prostate-specific antigen level was elevated (10.29 ng/mL). Also, the radiologist raised the possibility of retroperitoneal sarcoma based on the location and shape of the CT, and recurrence of lymphoma was also suspected based on the history of previous lymphoma. However, he had no systemic symptoms such as fever, weight loss, lymphadenopathy, or general weakness. So, prostate cancer metastasis was thought to be a more likely diagnosis.

To evaluate the mass, for the staging of the disease, and as a baseline for monitoring the treatment response, ^18^F-fluorodeoxyglucose (FDG) positron emission tomography/CT (PET/CT) was performed. Due to the purchase cost of the generator that produces ^68^Ga, ^68^Ga-PSMA-11 PET/CT is not yet possible at our hospital. Although prostate cancer metastasis was the most suspected differential diagnosis on the abdominal CT, we also had to differentiate between the possibility of sarcoma or recurrent lymphoma, so we performed ^18^F-FDG PET/CT first. MIP PET (A), axial CT (B–D), corresponding PET (E–G), and fused PET/CT images (H–J) showed a markedly metabolically active soft tissue mass in the distal ileum with invading right psoas muscle. Simultaneously, several hypermetabolic soft tissue nodules in the right retroperitoneal fascia and hypermetabolic mass along the right psoas muscle were also detected. In addition, there was left inguinal hernia (Figure 2). The findings were considered highly suspicious of malignancies such as recurrent lymphoma or soft tissue sarcoma. To confirm the diagnosis, surgical resection of the ileal mass was performed and histopathological and immunohistochemical examinations finally showed histiocytic sarcoma (HS) (Figure 3). The patient has been treated with four cycles of CHOP. The follow-up abdominal CT after three cycles showed a stable disease. A follow-up ^18^F-FDG PET/CT has not yet been performed and is scheduled to be performed after six cycles.

HS is an extremely rare hematopoietic neoplasm derived from non-Langerhans histiocytic cells of the monocyte/macrophage system. HS can be isolated or associated with other hematological neoplasms like non-Hodgkin lymphoma, myelodysplasia, or acute leukemia. HS has variable clinical presentations and outcomes, ranging from localized diseases to multiple sites within a single system to life-threatening disseminated diseases. The clinical presentation of HS varies depending on the organs involved. More commonly, patients present with a palpable mass lesion, symptoms related to the compression of surrounding organs, or systemic complaints such as fever and weight loss. Due to the small number of cases and the clinical and pathological features of the disease, the diagnosis can be challenging. HS associated with other hematological neoplasms tends to be more aggressive than the isolated form. It may appear up to 17 years after the initial malignancy [1,2,3]. There are very few cases of extranodal HS with ^18^F-FDG PET/CT findings in the literature [4,5,6,7,8]. However, we are unaware of the ^18^F-FDG PET/CT findings in extranodal HS arising after large B-cell lymphoma. The proper recognition of HS is important because this neoplasm’s clinical presentation and morphologic appearance may lead to being misdiagnosed as other hematologic malignancies. We should realize that HS may occur during or after the successful treatment of lymphoblastic neoplasms.

## Figures and Tables

**Figure 1:**
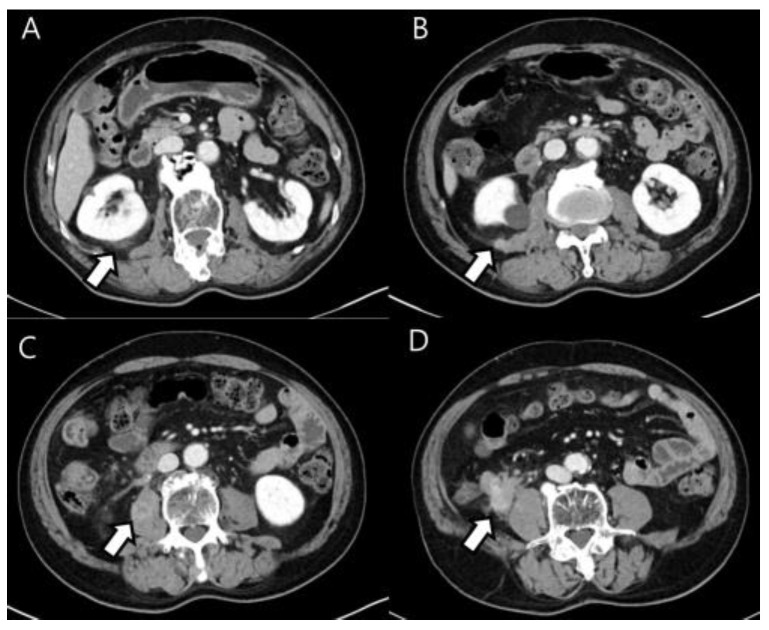
Axial, intravenous, contrast-enhanced CT scan of the abdomen (**A**–**D**) reveals an infiltrative-enhancing soft tissue mass in the terminal ileum. It also shows several enhanced soft tissue nodules in the right Gerota’s fascia, transversalis fascia, posterior pararenal fascia and right psoas muscle.

**Figure 2:**
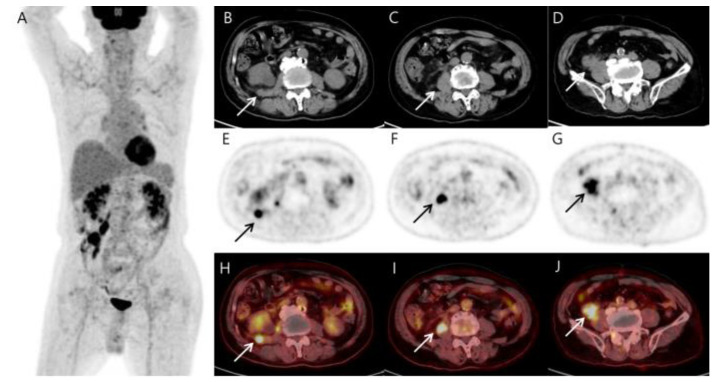
MIP (**A**), CT (**B**–**D**), PET (**E**–**G**), and fusion PET/CT (**H**–**J**) of the ^18^F-FDG PET/CT images reveal several hypermetabolic soft tissue nodules (arrows) involving the terminal ileum (SUVmax 17.87), right psoas muscle (SUVmax 14.87), and reptroperitoneum (SUVmax 12.22). The terminal ileal mass shows a comet-tail-like pattern of increasing absorption toward the back, suggesting invasion into the psoas muscle. Other than the above-described area, no significant abnormal findings in other parts of the body are included in the image. As shown in the MIP image, it differs too much from the typical prostate cancer metastasis that spreads from bottom to top along lymph nodes to be considered prostate cancer metastasis. Additionally, it is not appropriate to consider it a small intestinal adenocarcinoma and metastasis because the patient has no symptoms. The patient had previously been treated for lymphoma, and based on the ^18^F-FDG PET/CT findings, we first considered the possibility of recurrent lymphoma or sarcoma.

**Figure 3:**
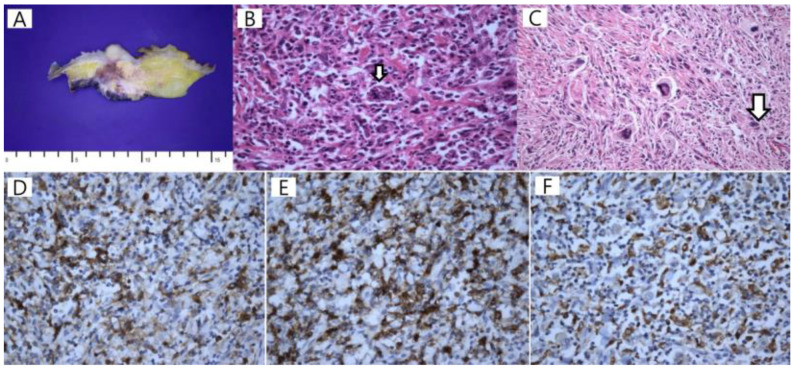
An infiltrative mass with mucosal irregularity was observed in the ileum on the gross examination of the excised ileal mass. The tumor involved mucosa to the serosa, and the cut surface was pale yellowish white, firm, and fibrotic with multifocal hemorrhage (**A**). Tumor cells in the background of the inflammatory cell infiltration had large oval nuclei with vesicular chromatin and prominent nucleoli. Some tumor cells show hemophagocytosis and emperipolesis ((**B**), H & E ×400, white arrow). Multinucleated giant tumor cells were also found ((**C**), H & E ×200, white arrow). Immunohistochemical staining (**D**–**F**) revealed positive CD4 ((**D**), ×400), CD45 ((**E**), ×400), and CD68 ((**F**), ×400). These findings were compatible with the diagnosis of histiocytic sarcoma (HS).

## Data Availability

The data that support the findings of this study are available from the corresponding author M.C., upon reasonable request.
